# A sustainable protocol for the synthesis of *N*-acyl tryptamines, a class of potential gut microbiota-derived endocannabinoid-like mediators

**DOI:** 10.3389/fchem.2024.1436008

**Published:** 2024-10-10

**Authors:** Rosaria Villano, Vincenzo Di Marzo

**Affiliations:** ^1^ Istituto di Chimica Biomolecolare, Consiglio Nazionale delle Ricerche, Pozzuoli, Italy; ^2^ Canada Excellence Research Chair on the Microbiome-Endocannabinoidome Axis in Metabolic Health, Faculty of Medicine and Faculty of Agricultural and Food Sciences, Centre de Recherche de l’Institut de Cardiologie et Pneumologie de l’Université et Institut sur la Nutrition et les Aliments Fonctionnels, Centre NUTRISS, Université Laval, Quebec, Canada

**Keywords:** *N*-acyl tryptamines, microbiota, green chemistry, organic synthesis, propylphosphonic anhydride

## Abstract

A simple and sustainable propylphosphonic anhydride (T3P)-assisted methodology for the synthesis of *N*-acyl tryptamines, an interesting class of gut microbiota-derived endocannabinoid-like lipid mediators, was proposed. This protocol is characterized by great operational simplicity, and all products were obtained at room temperature, without the use of an inert atmosphere and by using limited amounts of non-halogenated solvents. Finally, the possibility to realize the reaction under mechanochemical conditions was explored with interesting results.

## Graphical Abstract

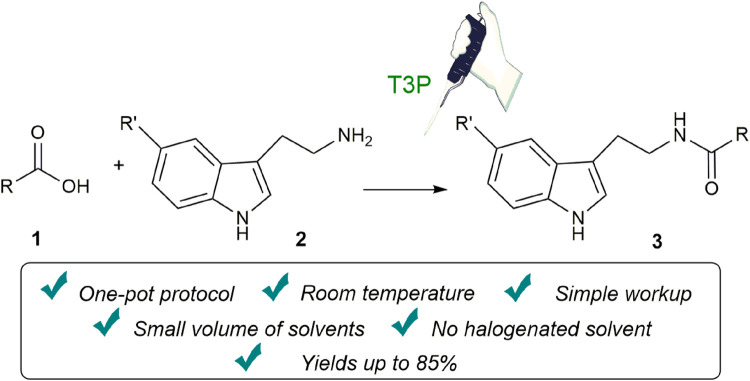

## 1 Introduction

Tryptamine is a metabolite of tryptophan produced by the gut microbiota through the indole pathway (Roth et al., 2021). Recently, it was demonstrated that the gut microbiota can also biosynthesize *N*-acyl tryptamines, i.e., compounds in which tryptamine is linked to bioavailable endogenous/dietary fatty acids (Chang et al., 2021). These amides, from a structural point of view, are very similar to endogenous signaling molecules belonging to the endocannabinoidome (Di Marzo, 2020) [e.g., *N*-acyl serotonins (Verhoeckx et al., 2011)], and, as such, they often compete for the same molecular targets, thus resulting bioactive (Chang et al., 2021; Ki et al., 2020).

Considering the interesting bioactivities that are emerging for *N*-acyl tryptamines, the design of new synthetic strategies (Nicolaou, 2014; Villano et al., 2022) is fundamental in order to have sufficient quantities of these molecules to test with the aim of 1) extending the screening of their bioactivities and understanding their role and mechanisms of action and 2) inserting appropriate structural modifications in the original scaffold for structure–activity relationship (SAR) studies. In this context, the development of new sustainable methodologies, based on the use of low-toxicity reagents, that maximize the quantity of the product and reduce the quantity of waste, as an alternative to obsolete high-impact methodologies, represents the real challenge (Horváth and Anastas, 2007; Li and Trost, 2008).

**SCHEME 1:**
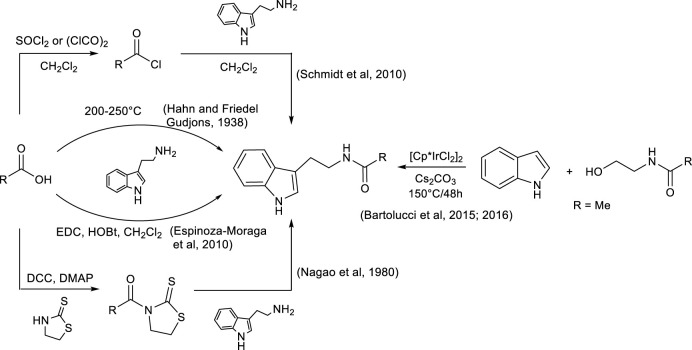
Some synthetic strategies reported in the literature for the production of *N*-acyl tryptamines.

Some methods for the synthesis of *N*-acyl tryptamines are reported in the literature (Scheme 1): their synthesis was carried out at high temperatures (approximately 200°C) with complex reaction mixtures being produced. Thus, difficult purification was often required, and the method was not suitable for thermolabile products or carboxylic acids (Hahn and Friedel Gudjons, 1938); alternatively, carboxylic acid could be converted into acyl chloride with SOCl_2_ or oxalyl chloride before a subsequent reaction with tryptamine. However, this alternative method is not very sustainable due to the use of toxic chlorinating agents and, often, halogenated solvents, especially during acyl halide synthesis (Schmidt et al., 2010).

More recently, *N*-acetyl tryptamines were synthesized by the direct alkylation of indoles with *N*-acetylethanolamine, using [Cp*IrCl_2_]_2_ and Cs_2_CO_3_ at high temperature; the corresponding products were isolated with satisfactory yields, although the methodology was limited to the production of acetyl derivatives (Bartolucci et al., 2015; Bartolucci et al., 2016).

Some coupling reagents, such as DCC/thiazolidine-2-thione (Nagao et al., 1980) or the EDC/HOBt (Espinoza-Moraga et al., 2010) system, were also used to promote the reaction between carboxylic acid and tryptamine, but, even in this case, despite good yields, the coupling reagents are not sustainable. DCC and EDC are substances characterized by acute toxicity and can cause organ damage (Jordan et al., 2021), while some additives such as HOBt, in addition to being harmful to health, show explosive properties at high temperatures or under mechanical stress (Wehrstedt et al., 2005), and therefore, many precautions must be taken during their use and storage. Furthermore, the reactions promoted by DCC or EDC are often carried out in halogenated solvents and at high dilutions, conditions that further reduce the sustainability of the process. Finally, another problem associated with the use of these classical coupling reagents is that their by-products are insoluble in water but show partial solubility in organic solvents, and therefore, during the extraction step, they remain in the mixture with the reaction product. Consequently, laborious chromatographic purifications are often required to obtain the pure reaction product, with a large increase in the volume of solvents, chromatographic materials, and energy used.

Propylphosphonic anhydride (T3P) (Basavaprabhu et al., 2013) is emerging as a good alternative to other less sustainable coupling reagents (such as the mentioned DCC and EDC); it was successfully used in peptide synthesis (Wissmann and Kleiner, 1980; Mattellone et al., 2023a; Mattellone et al., 2023b) even in the solid state (Al Musaimi et al., 2021) and, recently, also in the reaction between tryptamine and carboxylic acids (Ábrányi-Balogh et al., 2016). However, in the latter case, the experimental conditions (no base, MW irradiation, 120°C–150°C, and high pressure) did not allow the isolation of *N*-acyl tryptamines but favored the synthesis of 1-substituted-3,4-dihydro-β-carbolines *via* a cascade mechanism (acylation of tryptamine and subsequent Bischler–Napieralski cyclization of the amide, in a consecutive and one-pot manner).

Based on the indications of the ACS Green Chemistry Institute (Isidro-Llobet et al., 2019), T3P is considered a green reagent, thanks to its stability and low-toxicity/allergy profile, characteristics that make it an excellent candidate for the development of sustainable methodologies. Furthermore, it is commercially available in solution with different organic solvents (EtOAc, 2-MeTHF, etc.), and these formulations increase its stability and also make it suitable for use in small-scale reactions. Finally, unlike as with other classic coupling reagents (e.g., DCC and EDC), T3P by-products are soluble in water, and therefore, at the end of the reaction, they can be easily separated from the reaction mixture by simple aqueous extraction (Davidson et al., 2021), thus making the subsequent purification phase particularly simple while significantly reducing the volume of organic solvents and chromatographic materials. These aspects are particularly interesting, considering that the purification step often represents the most critical phase for the sustainability of the process (Duarte et al., 2015).

In view of this background and the biological importance of *N*-acyl tryptamines, in this study, we report a simple one-pot methodology for the synthesis of this class of molecules, using T3P as a coupling reagent and minimal volumes of non-halogenated solvents (for the reaction, workup, and purification steps).

## 2 Methods

### 2.1 General

All reagents and solvents (HPLC grade) were used without further purification. Reactions were monitored by thin-layer chromatography (TLC) on Merck silica gel plates (0.25 mm) and visualized using UV light at 254 nm and a cerium sulfate reagent. Column chromatographic purification of products was carried out using silica gel 60 (particle size: 0.040–0.063 mm; Merck). ^1^H NMR and ^13^C NMR spectra were recorded on a Bruker DRX 600 spectrometer equipped with an inverse TCI CryoProbe at room temperature (RT) in CDCl_3_. Chemical shifts are reported in ppm, and multiplicities are indicated by s (singlet), d (doublet), t (triplet), q (quartet), m (multiplet), and br (broad). Coupling constants (J) are reported in Hz. Yields are given for isolated products showing one spot on a TLC plate and no impurities detectable in the NMR spectrum. Spectroscopic characterizations of products **3aa** (Schmidt et al., 2010), **3ba** (Schmidt et al., 2010), **3ea** (Sládeková et al., 2023), **3da** (Singh and Singh, 2009), **3eb** (Takao et al., 2015), and **3be** (Blickenstaff et al., 1994) matched those reported in the literature.

### 2.2 Synthetic procedures and characterization of products

#### 2.2.1 General procedure for the synthesis of *N*-acyl tryptamines **3**

In an 8-mL vial, tryptamine **2** (1.2 eq, 0.12 mmol), carboxylic acid **1** (0.1 mmol), Et_3_N (2.0 eq, 0.2 mmol), T3P (50 wt% solution in EtOAc, 1.5 eq, 0.15 mmol), and EtOAc (60 µL) were added. The resulting mixture was stirred for 24 h at room temperature, and then, it was quenched by the addition of H_2_O (2 mL) and EtOAc (2 mL). The phases were separated, and the H_2_O phase was extracted with EtOAc (2 × 2 mL). The combined organic phase was dried (Na_2_SO_4_) and concentrated. The residue was purified using a short pad of silica gel (petroleum ether/EtOAc: 7/3–1/1) to yield products **3**. All new compounds were fully characterized.

#### 2.2.2 General procedure for the synthesis of *N*-acyl tryptamine **3aa** under mechanochemical conditions

In an 8-mL vial, tryptamine **2a** (1.2 eq, 0.12 mmol), carboxylic acid **1a** (0.1 mmol), Et_3_N (2.0 eq, 0.2 mmol), T3P (50 wt% solution in EtOAc, 1.5 eq, 0.15 mmol), and two stainless steel balls (diameter: 5 mm) were added. The resulting mixture was ground at 2,500 rpm for 10 min at RT using a vortex mixer (IKA Vortex Genius 3, speed 6), and then, the vial was left closed at RT for an additional 10 min before the usual workup with H_2_O (1 mL) and EtOAc (1 mL). The phases were separated, and the H_2_O phase was extracted with EtOAc (2 × 1 mL). The combined organic phase was dried (Na_2_SO_4_) and concentrated. The residue was purified using a short pad of silica gel (petroleum ether/EtOAc: 7/3–1/1) to yield product **3aa**.

*N-(2-(1H-indol-3-yl)ethyl)dodecanamide or N-lauroyl tryptamine (**3ca**)*. Prepared according to the general procedure from **1c** and **2a**. Colorless amorphous solid, 27.4 mg (0.08 mmol), and 80% yield. R_f_ value: 0.1 (petroleum ether/EtOAc: 7/3). ^1^H NMR (CDCl_3_, 600 MHz) δ: 8.1 (bs, 1H), 7.61 (d, J = 7.9 Hz, 1H), 7.38 (d, J = 8.2 Hz, 1H), 7.21 (t, J = 7.6 Hz, 1H), 7.13 (t, J = 7.6 Hz, 1H), 7.04 (bs, 1H), 5.48 (bs, 1H), 3.61 (q, J = 6.2 Hz, 2H), 2.98 (t, J = 6.7 Hz, 2H), 2.09 (t, J = 7.5 Hz, 2H), 1.61–1.54 (m, overlapping H_2_O residual peak, 2H), 1.31–1.21 (m, 16H), and 0.88 (t, J = 6.8 Hz, 3H). ^13^C NMR (CDCl_3_, 150 MHz) δ: 173.1, 136.4, 127.4, 122.3, 122.0, 119.5, 118.8, 113.2, 111.2, 39.6, 36.9, 31.9, 29.6, 29.5, 29.4, 29.3, 25.7, 25.4, 22.7, and 14.1.

*N-(2-(1H-indol-3-yl)ethyl)arachidonamide or N-arachidonoyl tryptamine (**3fa**)*. Prepared according to the general procedure from **1f** and **2a**. Pale yellow oil, 23.0 mg (0.055 mmol). R_f_ value: 0.1 (petroleum ether/EtOAc: 7/3). ^1^H NMR (CDCl_3_, 600 MHz) δ: 8.20 (bs, 1H), 7.61 (d, J = 7.9 Hz, 1H), 7.38 (d, J = 8.1 Hz, 1H), 7.21 (t, J = 7.2 Hz, 1H), 7.13 (t, J = 7.3 Hz, 1H), 7.03 (bs, 1H), 5.52 (bs, 1H), 5.42–5.31 (m, 8H), 3.61 (q, J = 6.6 Hz, 2H), 2.98 (t, J = 6.8 Hz, 2H), 2.85–2.76 (m, 6H), 2.14–2.02 (m, 6H), 1.68 (m, 2H), 1.38–1.26 (m, 6H), and 0.89 (t, J = 6.4 Hz, 3H). ^13^C NMR (CDCl_3_, 150 MHz) δ: 172.9, 136.4, 130.5, 129.2, 128.7, 128.6, 128.3, 128.2, 127.9, 127.5, 127.4, 122.2, 122.0, 119.5, 118.7, 113.0, 111.3, 39.7, 36.2, 31.5, 29.3, 27.2, 26.7, 25.6, 25.5, 25.4, 22.6, and 14.1.

*N-(2-(5-hydroxy-1H-indol-3-yl)ethyl)palmitamide or N-palmitoyl serotonin (**3bb**)*. Prepared according to the general procedure from **1b** and **2b**. Colorless amorphous solid, 15.6 mg (0.038 mmol). R_f_ value: 0.1 (petroleum ether/EtOAc: 7/3). ^1^H NMR (CDCl_3_, 600 MHz) δ: 7.88 (bs, 1H), 7.23 (d, J = 8.6 Hz, 1H), 7.04 (d, J = 2.3 Hz, 1H), 7.01 (d, J = 2.2 Hz, 1H), 6.79 (dd, J = 8.6, 2.4 Hz, 1H), 5.50 (bs, 1H), 4.89 (bs, 1H), 3.57 (q, J = 6.7 Hz, 2H), 2.90 (t, J = 6.9 Hz, 2H), 2.11 (t, J = 7.5 Hz, 2H), 1.60–1.55 (m, overlapping H_2_O residual peak, 2H), 1.31–1.21 (m, 24H), and 0.88 (t, J = 6.8 Hz, 3H). ^13^C NMR (CDCl_3_, 150 MHz) δ: 173.2, 149.6, 131.6, 128.1, 123.0, 112.5, 112.1, 111.9, 103.3, 39.5, 36.9, 31.9, 29.7, 29.6, 29.5, 29.4, 29.3, 25.8, 25.5, 22.7, and 14.1.

*N-(2-(5-methyl-1H-indol-3-yl)ethyl)palmitamide or N-palmitoyl 5-methyltryptamine (**3bc**)*. Prepared according to the general procedure from **1b** and **2c**. Colorless amorphous solid, 34.3 mg (0.083 mmol). R_f_ value: 0.1 (petroleum ether/EtOAc: 7/3). ^1^H NMR (CDCl_3_, 600 MHz) δ: 7.96 (bs, 1H), 7.39 (bs, 1H), 7.27 (d, overlapping solvent peak, 1H), 7.04 (d, J = 8.2 Hz, 1H), 6.99 (d, J = 2.0 Hz, 1H), 5.48 (bs, 1H), 3.59 (q, J = 6.5 Hz, 2H), 2.94 (t, J = 6.7, 2H), 2.46 (s, 3H), 2.09 (t, J = 7.5 Hz, 2H), 1.66–1.52 (m, overlapping H2O residual peak, 2H), 1.30–1.20 (m, 24H), and 0.88 (t, J = 6.8 Hz, 3H). ^13^C NMR (CDCl_3_, 150 MHz) δ: 173.1, 134.7, 128.8, 127.6, 123.9, 122.1, 118.4, 112.7, 110.9, 39.7, 37.0, 31.9, 29.7, 29.67, 29.63, 29.5, 29.4, 29.3, 25.8, 25.4, 22.7, 21.5, and 14.1.

*N-(2-(5-methyl-1H-indol-3-yl)ethyl)oleamide or N-oleoyl 5-methyltryptamine (* ***3ec*** *)*. Prepared according to the general procedure from **1e** and **2c**. Colorless wax, 35.1 mg (0.080 mmol). R_f_ value: 0.2 (petroleum ether/EtOAc: 7/3). ^1^H NMR (CDCl_3_, 600 MHz) δ: 8.25 (bs, 1H), 7.38 (bs, 1H), 7.26 ((d, J = 8.3 Hz, overlapping solvent peak, 1H), 7.03 (d, J = 8.2 Hz, 1H), 6.97 (bs, 1H), 5.58 (bs, 1H), 5.34 (m, 2H), 3.59 (q, J = 6.5 Hz, 2H), 2.94 (t, J = 6.7 Hz, 2H), 2.46 (s, 3H), 2.09 (t, J = 7.6 Hz, 2H), 2.01 (m, 4H), 1.58 (m, 2H), 1.36–1.23 (m, 20H), and 0.89 (t, J = 5.9 Hz, 3H). ^13^C NMR (CDCl_3_, 150 MHz) δ: 173.3, 134.8, 130.0, 129.8, 128.7, 127.6, 123.8, 122.2, 118.4, 112.4, 111.0, 39.8, 36.9, 31.9, 29.8, 29.7, 29.5, 29.3, 29.2, 27.24, 27.2, 25.8, 25.4, 22.7, 21.5, and 14.1.

*N-(2-(5-fluoro-1H-indol-3-yl)ethyl)palmitamide or N-palmitoyl 5-fluorotryptamine (**3bd**)*. Prepared according to the general procedure from **1b** and **2d**. Amorphous solid, 30.8 mg (0.074 mmol). R_f_ value: 0.1 (petroleum ether/EtOAc 7/3). ^1^H NMR (CDCl_3_, 600 MHz) δ: 8.08 (bs, 1H), 7.28 (dd, J = 8.8, 4.3 Hz, 1H), 7.23 (dd, J = 9.5, 2.3 Hz, 1H), 7.08 (d, J = 1.9 Hz, 1H), 6.95 (td, J = 9.0, 2.4 Hz, 1H), 5.48 (bs, 1H), 3.58 (q, J = 6.7 Hz, 2H), 2.92 (t, J = 6.7 Hz, 2H), 2.11 (t, J = 7.6 Hz, 2H), 1.60–1.55 (m, overlapping H_2_O residual peak, 2H), 1.29–1.23 (m, 24H), and 0.88 (t, J = 6.9 Hz, 3H). ^13^C NMR (CDCl_3_, 150 MHz) δ 173.2, 158.6, 157.0, 132.8, 127.8 (d, J_C-F_ = 9.6 Hz), 123.7, 113.4, 111.8 (d, J_C-F_ = 9.6 Hz), 110.6 (d, J_C-F_ = 26.2 Hz), 103.6 (d, J_C-F_ = 23.3 Hz), 39.5, 36.9, 31.9, 29.7, 29.66, 29.63, 29.5, 29.4, 29.3, 25.7, 25.4, 22.7, and 14.1.

*N-(2-(5-fluoro-1H-indol-3-yl)ethyl)oleamide or N-oleoyl 5-fluorotryptamine (**3ed**)*. Prepared according to the general procedure from **1e** and **2d**. Amorphous solid, 24.7 mg (0.056 mmol). R_f_ value: 0.1 (petroleum ether/EtOAc: 7/3). ^1^H NMR (CDCl_3_, 600 MHz) δ: 8.26 (bs, 1H), 7.28 (dd, J = 8.8, 4.3 Hz, 1H), 7.23 (bd, J = 9.5 Hz, 1H), 7.07 (bs, 1H), 6.95 (bt, J = 8.9 Hz, 1H), 5.52 (bs, 1H), 5.34 (m, 2H), 3.58 (q, J = 6.5 Hz, 2H), 2.92 (t, J = 6.7 Hz, 2H), 2.11 (t, J = 7.6 Hz, 2H), 2.00 (m, 4H), 1.60–1.55 (m, 2H), 1.34–1.23 (m, 20H), and 0.88 (t, J = 6.9 Hz, 3H). ^13^C NMR (CDCl_3_, 150 MHz) δ: 173.2, 158.6, 157.0, 132.9, 130.0, 129.8, 127.8 (d, J_C-F_ = 9.4 Hz), 123.8, 113.3, 111.9 (d, J_C-F_ = 9.4 Hz), 110.6 (d, J_C-F_ = 26.2 Hz), 103.6 (d, J_C-F_ = 23.3 Hz), 39.6, 36.9, 31.9, 29.8, 29.7, 29.5, 29.3, 29.2, 29.1, 27.2, 27.18, 25.7, 25.4, 22.7, and 14.1.

*N-(2-(5-methoxy-1H-indol-3-yl)ethyl)oleamide or N-oleoyl 5-methoxytryptamine (**3ee**)*. Prepared according to the general procedure from **1e** and **2e**. Amorphous solid, 33.8 mg (0.074 mmol). R_f_ value: 0.1 (petroleum ether/EtOAc: 7/3). ^1^H NMR (CDCl_3_, 600 MHz) δ: 8.11 (bs, 1H), 7.26 (d, J = 8.7 Hz, overlapping solvent peak, 1H), 7.03 (d, J = 2.3 Hz, 1H), 7.00 (bs, 1H), 6.87 (dd, J = 8.8, 2.3 Hz, 1H), 5.53 (bs, 1H), 5.34 (m, 2H), 3.86 (s, 3H), 3.60 (q, J = 6.5 Hz, 2H), 2.94 (t, J = 6.7, 2H), 2.10 (t, J = 7.5 Hz, 2H), 2.00 (m, 4H), 1.60–1.55 (m, 2H), 1.35–1.23 (m, 20H), and 0.88 (t, J = 6.8 Hz, 3H). ^13^C NMR (CDCl_3_, 150 MHz) δ: 173.2, 154.1, 131.5, 130.0, 129.8, 127.7, 122.8, 112.8, 112.4, 112.0, 100.5, 55.9, 39.5, 36.9, 31.9, 29.8,29.7, 29.5, 29.3, 29.28, 29.1, 27.2, 27.19, 25.8, 25.4, 22.7, and 14.1.

*N-(2-(5-chloro-1H-indol-3-yl)ethyl)palmitamide or N-palmitoyl 5-chlorotryptamine (**3bf**)*. Prepared according to the general procedure from **1b** and **2f**. Amorphous solid, 31.0 mg (0.072 mmol). R_f_ value: 0.1 (petroleum ether/EtOAc: 7/3). ^1^H NMR (CDCl_3_, 600 MHz) δ: 8.12 (bs, 1H), 7.56 (d, J = 1.6 Hz, 1H), 7.29 (d, J = 8.6 Hz, 1H), 7.15 (dd, J = 8.6, 1.9 Hz, 1H), 7.06 (bd, J = 1.9 Hz, 1H), 5.47 (bs, 1H), 3.58 (q, J = 6.7 Hz, 2H), 2.93 (t, J = 6.7 Hz, 2H), 2.11 (t, J = 7.5 Hz, 2H), 1.60–1.55 (m, overlapping H_2_O residual peak, 2H), 1.30–1.23 (m, 24H), and 0.88 (t, J = 6.8 Hz, 3H). ^13^C NMR (CDCl_3_, 150 MHz) δ: 173.2, 134.7, 128.6, 125.3, 123.3, 122.5, 118.3, 113.1, 112.2, 39.6, 36.9, 31.9, 29.7, 29.66, 29.63, 29.5, 29.4, 29.3, 25.7, 25.3, 22.7, and 14.1.

*N-(2-(5-chloro-1H-indol-3-yl)ethyl)oleamide or N-oleoyl 5-chlorotryptamine (**3ef**)*. Prepared according to the general procedure from **1e** and **2f**. Colorless oil, 35.1 mg (0.076 mmol). R_f_ value: 0.1 (petroleum ether/EtOAc: 7/3). ^1^H NMR (CDCl_3_, 600 MHz) δ: 8.51 (bs, 1H), 7.54 (bs, 1H), 7.27 (bd, J = 8.5 Hz, 1H), 7.13 (m, 1H), 7.02 (m, 1H), 5.58 (bs, 1H), 5.34 (m, 2H), 3.57 (q, J = 6.0 Hz, 2H), 2.91 (t, J = 6.2 Hz, 2H), 2.12 (t, J = 7.6 Hz, 2H), 2.00 (m, 4H), 1.61–1.55 (m, 2H), 1.35–1.23 (m, 20H), and 0.87 (t, J = 6.8 Hz, 3H). ^13^C NMR (CDCl_3_, 150 MHz) δ: 173.3, 134.7, 130.0, 129.8, 128.5, 125.1, 123.5, 122.4, 118.2, 112.3, 39.8, 36.9, 31.9, 29.8, 29.7, 29.5, 29.3, 29.2, 29.1, 27.2, 27.19, 25.8, 25.3, 22.7, and 14.1.

## 3 Results and discussion

**SCHEME 2:**
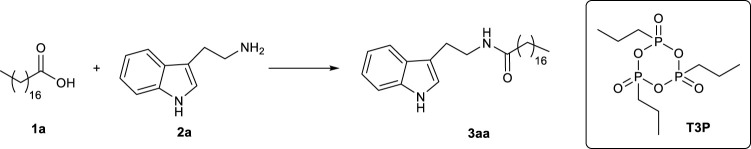
Model reaction of stearic acid **1a** with tryptamine **2a**.

In a preliminary study, stearic acid **1a** (0.1 mmol) was chosen as the model substrate and subjected to a coupling reaction with tryptamine **2a** (Scheme 2). The reaction was initially carried out for 24 h at RT, using 1 eq of T3P (commercially available as a 50 wt% solution in EtOAc) and adding a further small volume of EtOAc (60 µL) in order to solubilize the solid reagents and allow homogeneous mixing. The procedure was one-pot; hence, T3P was added together with the other reagents, without acid preactivation. Under these conditions, **3aa** was obtained only in trace amounts (entry 1, Table 1), and a slight improvement in yield was observed using a small excess of tryptamine (entry 2, Table 1).

**TABLE 1:** Optimization of the T3P-assisted coupling reaction between stearic acid 1a and tryptamine 2a.

Entry	2a (eq)	Time (h)/T (°C)	Et_3_N (eq)	T3P (eq)	Yield 3aa (mol%)^a^
1	1	24 h/rt	-	1	Trace
2	1.2	24 h/rt	-	1	25
3	1.2	24 h/rt	2	1	64
*4*	*1.2*	*24* * * *h/rt*	*2*	*1.5*	*83*
5	1.2	24 h/rt	1	1.5	65
6	1.2	24 h/rt	3	1.5	76
7	1.2	6 h/rt	2	1.5	50
8	1.2	48 h/rt	2	1.5	84

^a^ All the yields refer to isolated chromatographically pure compounds.

**SCHEME 3:**
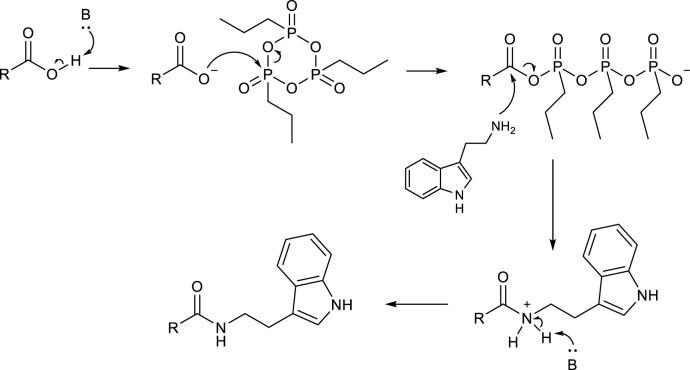
Proposed reaction mechanism.

In agreement with the hypothesized mechanism (Scheme 3), a clear improvement in the reaction yield was observed by adding 2 eq of a base (Et_3_N), which was necessary to deprotonate carboxylic acid and start the reaction mechanism (entry 3). An excess of T3P significantly improved the efficiency of the reaction (entry 4), while no further improvement was observed by using a smaller (entry 5) or a larger (entry 6) amount of base, of which 2 eq was the optimal quantity. Finally, 24 h was necessary to obtain high yields of product **3aa** (compare entry 4 with entries 7 and 8 in Table 1).

In order to test the general validity of the developed methodology, several carboxylic acids were submitted to the protocol using the optimized reaction conditions given in Table 1, entry 4.

**SCHEME 4:**
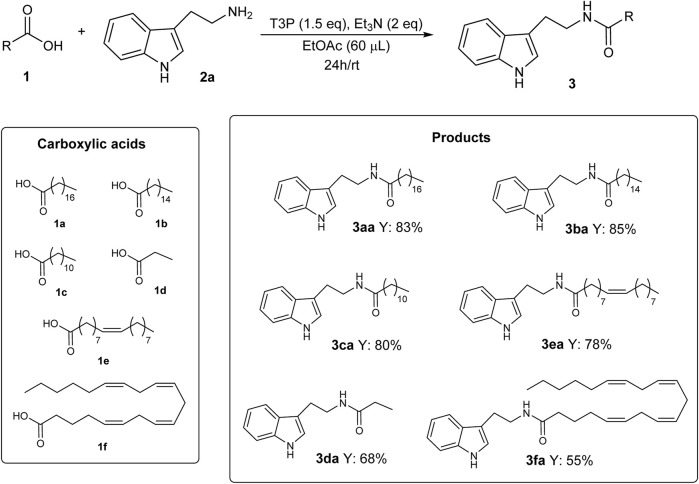
Substrate scope for the synthesis of *N*-acyl tryptamine **3**.

As shown in Scheme 4, good results were obtained in 24 h at RT with short-, medium-, and long-chain (saturated, monounsaturated, and polyunsaturated) carboxylic acids, confirming the validity and generality of the developed methodology.

**SCHEME 5:**
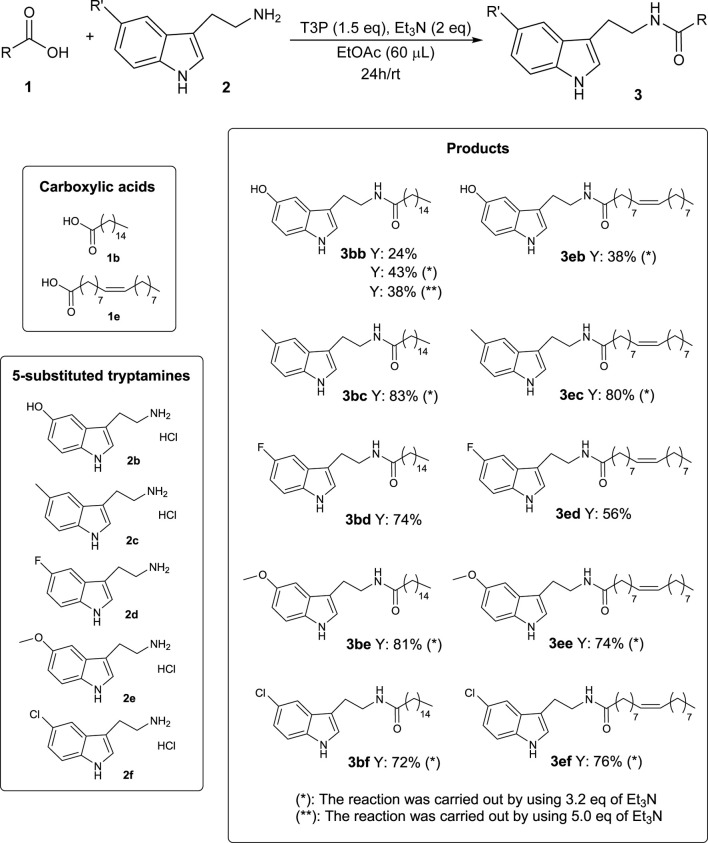
Substrate screening for the synthesis of *N*-acyl tryptamine **3**.

With the aim of extending the scope of the reaction, two bioavailable long-chain carboxylic acids (palmitic acid **1b** and oleic acid **1e**) were chosen as representative substrates, and the reaction was repeated with some tryptamine derivatives **2** exhibiting a substituent Rʹ at the 5-position (Scheme 5).

As shown in Scheme 5, most of the products were isolated with satisfactory yields. The worst efficiencies were obtained with serotonin **2b**, which was probably due to the interference of the unprotected phenolic OH with T3P and, consequently, with the reaction mechanism.

These data further confirm the validity and generality of the developed methodology, which also provided interesting results using 5-substituted tryptamines **2**, thus also allowing easy access to other classes of bioactive molecules, such as long-chain *N*-acyl serotonins (Ortar et al., 2007) (**3bb** and **3eb**) or melatonins (Blickenstaff et al., 1994) (**3be** and **3ee**).

It is important to note that, when using the 5-substituted tryptamine derivative **2** commercially available as hydrochloride salt (**2b**, **2c**, **2e**, and **2f**), the best yields were obtained using 3.2 eq of Et_3_N instead of 2 eq, in order to balance the HCl derived from the starting material (as shown for the product **3bb** in Scheme 5).

The impact of this new methodology for the synthesis of *N*-acyl tryptamines **3** can be better described by considering the importance that some products reported in this study have already proven to have. For example, *N*-lauroyl tryptamine **3ca** (Scheme 4) was found to not only activate serotonin receptors but also inhibit EBI2 (or GPR183), a receptor associated with inflammatory bowel disease (Chang et al., 2021). *N*-oleoyl tryptamine **3ea** (Scheme 4) was found to activate the pregnane X receptor (PXR), which is seemingly involved in several physiological responses (regulation of immune response, embryogenesis, metabolic disorders, etc.) (Sládeková et al., 2023). Furthermore, the bioactivities of some products given in Scheme 5 are described in the literature; for example, *N*-oleoyl serotonin **3eb** inhibits capsaicin-induced TRPV1 channel activation (Ortar et al., 2007); *N*-palmitoyl serotonin **3bb** inhibits L-dopa-induced dyskinesia in the mouse Parkinson model (Park et al., 2016); finally, for homologs of melatonin, such as *N*-palmitoyl 5-methoxytryptamine **3be**, radioprotective activity was highlighted (Blickenstaff et al., 1994).

**SCHEME 6:**
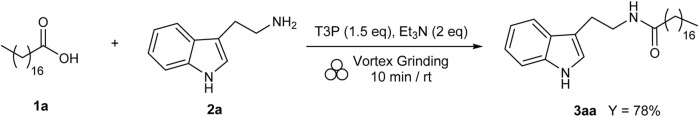
Synthesis of *N*-stearoyl tryptamine **3aa** under mechanochemical conditions.

Finally, the model reaction between stearic acid **1a** and tryptamine **2a** was repeated under mechanochemical conditions (Fantozzi et al., 2023) (Scheme 6). Mechanochemistry explores the possibility of inducing chemical reactions through mechanical forces, and recently, the IUPAC included it in the list of 10 chemical innovations that could change the world (https://www.chemistryworld.com/news/iupac-names-10-chemistry-innovations-that-will-change-the-world/3010335.article). In 2012, Stojakovic et al. (Stojakovic et al., 2012) demonstrated that an automated grinding method based on a vortex mixer could replace manual mortar-and-pestle grinding (a time-consuming and impractical procedure) and allow for the realization of processes under mechanochemical conditions.

When using this approach, all solid reagents were added to a transparent borosilicate vial with two stainless steel beads (diameter: 5 mm) in the presence of T3P (1.5 eq) and Et_3_N (2 eq), without adding a further solvent, and the non-homogeneous mixture was mixed for 10 min at RT using a vortex mixer (commonly used for the preparation of biological samples).

At the end of the process, the mixture appeared homogeneous, thanks to the mechanical stress produced by the grinding in the vortex due to the collisions among reagents, beads, and vial. This alternative procedure provided product **3aa** in only few minutes with a yield of 78%, comparable to the yield obtained with the conventional methodology under the optimized conditions (83% in 24 h/RT, as reported in entry 4, Table 1), with a significant reduction in reaction times and energy consumption.

To the best of our knowledge, this is the first synthesis of *N*-stearoyl tryptamine **3aa** under mechanochemical conditions. Starting from this very encouraging preliminary result, further experiments for the development of a general mechanochemical methodology for *N*-acyl tryptamines **3** are in progress. In particular, a scrupulous screening of the experimental conditions will be carried out, with the aim of reducing excess reagents and improving the yields and “greenness” associated with the methodology, further considering the biological importance of the products with this scaffold and the need to elaborate new sustainable protocols for their production. For this purpose, more sustainable bases will also be tested as an alternative to Et_3_N, and the best reaction conditions will finally be applied to the reaction with different carboxylic acids **1** and tryptamine derivatives **2**, using alternative milling devices.

## 4 Conclusions

In conclusion, a simple and effective methodology for the synthesis of an interesting class of gut microbiota-derived endocannabinoid-like lipid mediators, called *N*-acyl tryptamines, was proposed. The reaction was promoted by T3P, a sustainable coupling reagent that allowed the realization of a one-pot process, without preactivation of carboxylic acid. A key advantage of this methodology was the use of limited amounts of organic solvents in each phase of the process (reaction, workup, and purification) and no halogenated solvent. Furthermore, the reactions were carried out at room temperature, without the use of an inert atmosphere. The by-products of T3P were highly soluble in water, so they could be completely removed from the reaction mixture by a simple aqueous extraction, thus also making the subsequent purification phase particularly simple and with reduced environmental impact. Finally, the possibility of carrying out the reaction under mechanochemical conditions was explored with interesting prospects for a future development, thanks to the drastic reduction in reaction times and energy consumption.

## Data Availability

The original contributions presented in the study are included in the article further inquiries can be directed to the corresponding author.
